# Histology and μCT reveal the unique evolution and development of multiple tooth rows in the synapsid *Endothiodon*

**DOI:** 10.1038/s41598-021-95993-6

**Published:** 2021-08-19

**Authors:** Savannah L. Olroyd, Aaron R. H. LeBlanc, Ricardo Araújo, Kenneth D. Angielczyk, Aliénor Duhamel, Julien Benoit, Marisa Amaral

**Affiliations:** 1 Department of Biology, University of Washington, Seattle, USA; 2 Centre for Oral, Clinical & Translational Sciences, King’s College London, London, UK; 3 Instituto de Plasmas e Fusão Nuclear, Universidade de Lisboa, Lisbon, Portugal; 4 Negaunee Integrative Research Center, Field Museum of Natural History, Chicago, USA; 5 Evolutionary Studies Institute, School of Geosciences, University of the Witwatersrand, Johannesburg, South Africa; 6 Museu Nacional de Geologia, Maputo, Mozambique

**Keywords:** Palaeontology, X-ray tomography, Bone development

## Abstract

Several amniote lineages independently evolved multiple rows of marginal teeth in response to the challenge of processing high fiber plant matter. Multiple tooth rows develop via alterations to tooth replacement in captorhinid reptiles and ornithischian dinosaurs, but the specific changes that produce this morphology differ, reflecting differences in their modes of tooth attachment. To further understand the mechanisms by which multiple tooth rows can develop, we examined this feature in *Endothiodon bathystoma*, a member of the only synapsid clade (Anomodontia) to evolve a multi-rowed marginal dentition. We histologically sampled *Endothiodon* mandibles with and without multiple tooth rows as well as single-rowed maxillae. We also segmented functional and replacement teeth in µ-CT scanned mandibles and maxillae of *Endothiodon* and several other anomodonts with ‘postcanine’ teeth to characterize tooth replacement in the clade. All anomodonts in our sample displayed a space around the tooth roots for a soft tissue attachment between tooth and jaw in life. Trails of alveolar bone indicate varying degrees of labial migration of teeth through ontogeny, often altering the spatial relationships of functional and replacement teeth in the upper and lower jaws. We present a model of multiple tooth row development in *E. bathystoma* in which labial migration of functional teeth was extensive enough to prevent resorption and replacement by newer generations of teeth. This model represents another mechanism by which multiple tooth rows evolved in amniotes. The multiple tooth rows of *E. bathystoma* may have provided more extensive contact between the teeth and a triturating surface on the palatine during chewing.

## Introduction

Mammal teeth are well-adapted to oral processing and dental occlusion, with individual teeth forming elaborate structures for crushing, puncturing, and grinding tough foods against their counterparts in the opposing jaws^1^. Structurally complex, wear-resistant teeth like those of many mammals are seldom seen in non-mammalian amniotes. Most extant reptile teeth, for example, are frequently replaced, structurally simpler, and often are not used for the same level of sophisticated oral processing^2^. A simple dentition can limit the ecological niches that non-mammalian amniotes may exploit, particularly ones involving ingestion of high-fiber plant matter. However, the amniote fossil record has revealed several examples of innovative solutions to the problem of oral processing that do not exist today, the most impressive of which are the dental batteries of several herbivorous and omnivorous taxa.

Some polyphyodont amniotes independently evolved multiple rows of horizontally-aligned or vertically-stacked teeth that were continually replenished with new teeth as the old ones wore away^3^. These dentition-level adaptations resulted from evolutionary changes in the architecture and growth of the jaws and the process of tooth replacement, allowing older teeth to be retained in the mouth rather than being shed. The most studied dental batteries are found in Permian captorhinid reptiles and Late Cretaceous ornithischian dinosaurs. These two examples are remarkably different in their development. In captorhinids, new teeth quickly fused to the underlying jawbone and were passively carried labially along the jaws through a novel form of jaw growth, forming a slowly drifting conveyor belt of teeth^4–6^. By comparison, hadrosaurid and ceratopsid dinosaur teeth formed along the bases of vertical stacks of teeth and were carried towards the grinding surfaces through continual tooth eruption and a sophisticated network of ligaments that anchored the teeth together within the battery^7–9^. Although the mechanisms through which different amniotes maintained their dental batteries may differ, studying these unusual structures offers rare windows into the modularity and lability of the amniote dentition and how non-mammalian amniotes adapted to abrasive diets.

**Figure 1:**
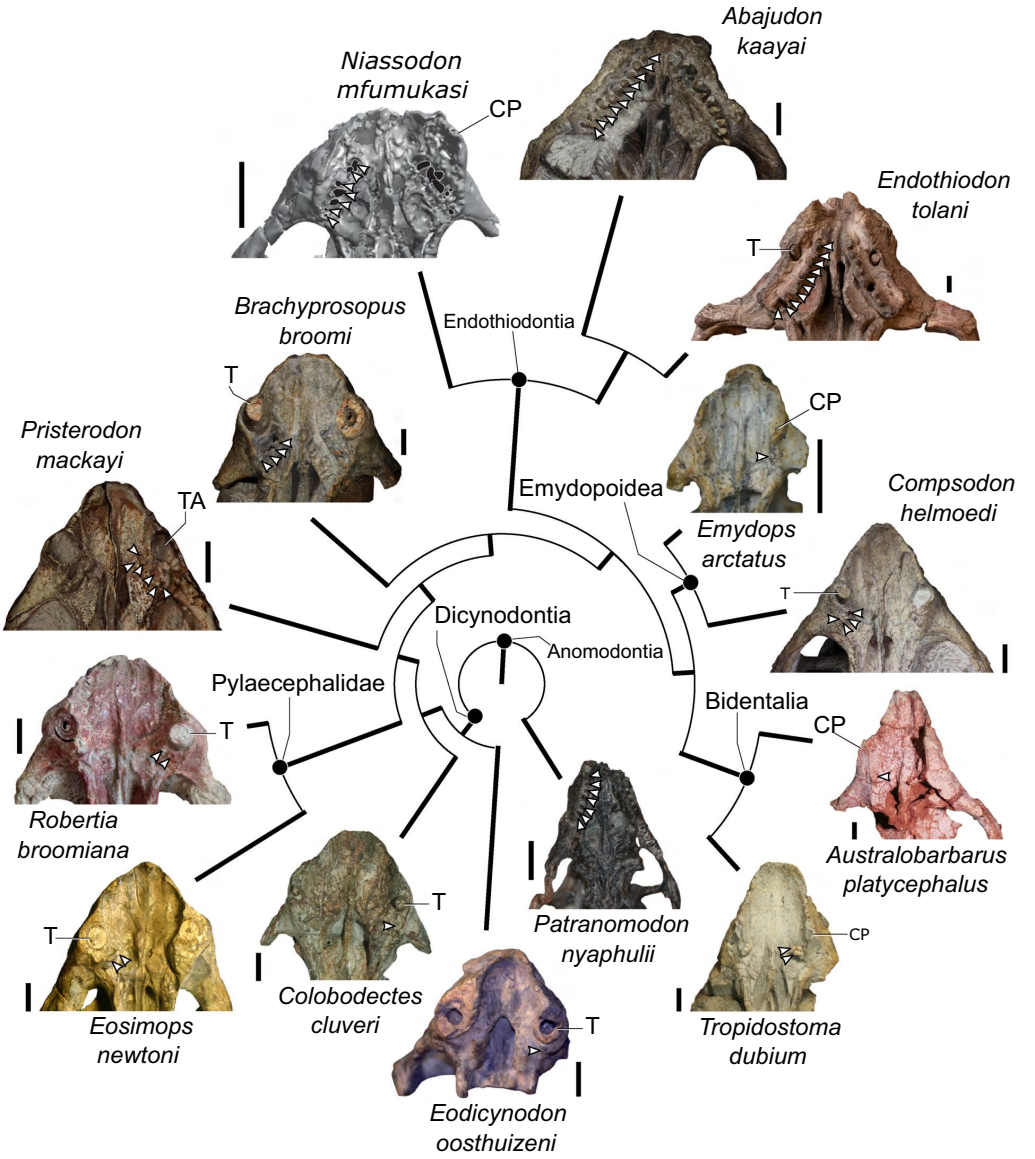
Simplified phylogeny showing different arrangements of the upper ‘postcanine’ teeth of dicynodonts; arrows indicate teeth or open alveoli. ‘Postcanines’ are typically located in the maxilla, although the anteriormost two teeth of *Abajudon* and *Endothiodon* are located in the premaxilla. Phylogeny based on Olroyd et al.^47^, Kammerer and Ordoñez^106^, and Angielczyk et al.^107^, but note that other topologies for *Pristerodon, Brachyprosopus*, the endothiodonts, and the emydopoids have appeared in recent phylogenetic analyses^42,108,109^. Branch lengths are arbitrary. Scale bars are 1 cm. Specimens: *Patranomodon nyaphulii* NMQR 3000; *Eodicynodon oosthuizeni* NMQR 2909; *Colobodectes cluveri* NMQR 3329; *Eosimops newtoni* USNM 23345; *Robertia broomiana* SAM-PK-K7807; *Pristerodon mackayi* NHCC LB355; *Brachyprosopus broomi* FMNH UR 2490; *Abajudon kaayai* NHCC LB314; *Endothiodon tolani* NHMUK PV R12443; *Emydops acrtatus* SAM-PK-11060; *Compsodon helmoedi* NHCC LB14; *Tropidostoma dubium* CGP/1/2173; *Australobarbarus platycephalus* PIN 4678/3. *CP* caniniform process, *T* tusk, *TA* tusk alveolus.

One intriguing example that warrants more in-depth study is the multiple-tooth-rowed dentition of the extinct anomodont *Endothiodon*^10,11^*.* Though multiple tooth rows are also seen in the anomodonts *Biseridens*^12^ and *Ulemica*^13,14^, the presence of such a complex dentition in *Endothiodon* is especially interesting because it is part of a clade (Dicynodontia) that is known for its reduced dentition (Fig. 1). Dicynodontia, the major subclade of Anomodontia, is an important group of mostly herbivorous non-mammalian therapsids known from the Permian and Triassic periods of Earth history (e.g., see review^15^). The clade is particularly noteworthy for its species richness^16^, high relative abundance in many late Permian and Early Triassic assemblages^17–19^, global distribution^20^, and survival of the Permo-Triassic mass extinction^21^. Compared to other therapsids, the dicynodont feeding apparatus is highly transformed to accommodate their mostly herbivorous diet^13,22–26^. Perhaps the most striking feature is the presence of an edentulous bony beak and an associated keratinous rhamphotheca^27–31^. Not all dicynodonts are completely toothless, however. In addition to the maxillary caniniform tusks found in many dicynodonts, several Permian species retain some ‘postcanine’ teeth (sensu Fröbisch and Reisz^32^) in the maxilla and dentary, and a few also possess a small number of premaxillary teeth.

There are two basic arrangements of ‘postcanine’ teeth found among dicynodonts (Fig. 1). In the first arrangement, a small number of teeth are located relatively far laterally on the maxilla, posterior to the caniniform process of the maxilla and the tusk (when present). This state is typical of the stemward dicynodonts *Eodicynodon* (though the presence of ‘postcanines’ in this genus is variable)^33,34^, *Colobodectes*^35,36^, and *Lanthanostegus*^37,38^, as well as the later-diverging emydopoids *Emydops*^32^ and *Compsodon*^15^. The second arrangement consists of an anteromedially to posterolaterally-trending row of teeth on the maxilla, with at least the anterior end of the tooth row located medial to the caniniform process and the tusk (when present). This condition is widely distributed among Permian dicynodonts, including the toothed pylaecephalids *Eosimops*, *Robertia*, and *Prosictodon*^33,39–41^, the stemward dicynodonts *Pristerodon* and *Brachyprosopus*^33,39,42,43^, the endothiodonts *Endothiodon*, *Niassodon*, and *Abajudon*^43–47^, and the cryptodonts *Tropidostoma* and *Australobarbarus*^33,48–50^. The number of teeth and the corresponding length of the tooth row is variable both intraspecifically^43,51,52^ and interspecifically, with the longest tooth rows found in the endothiodonts, in which the anteriormost teeth are actually located on the premaxilla^44,45,47^. Mandibular teeth in dicynodonts are consistently located towards the medial side of the dentary, although the length of the tooth row and its position relative to other anatomical landmarks such as the dentary table and posterior dentary sulcus are variable^33,39,41^.

*Endothiodon bathystoma* was the first dicynodont with ‘postcanine’ teeth to be described, and in his initial description, Owen^53^ stated that two or more rows of irregularly-placed teeth were present in the skull, and three rows of teeth were present in the mandible. Subsequently, Owen^54^ described a second species of *Endothiodon*, *E. uniseries*, which he in part differentiated from *E. bathystoma* based on the presence of a single row of upper teeth. Cox^44^ questioned the presence of multiple rows of teeth in the maxillae of *E. bathystoma*, instead suggesting that some of the teeth Owen identified as upper teeth were actually displaced lower teeth preserved on the palate. The nature of tooth replacement in *Endothiodon* and its relationship to the organization of the ‘postcanines’ was only briefly considered by Cox^44^, but he did note that replacement teeth in the mandible erupted lingual to older functional teeth. Latimer et al.^55^ conducted a detailed examination of tooth replacement patterns in *Endothiodon* using a series of specimens collected in the Metangula Graben of Mozambique. They concluded that the single row of mandibular teeth was organized into obliquely-oriented Zahnreihen, or replacement waves, that resulted in an appearance similar to multiple longitudinally-oriented rows of teeth. They also suggested that the single row of teeth present in the maxilla may have been replaced in a Zahnreihen-like fashion, but the available material did not allow them to determine this with certainty. Most descriptive and taxonomic work on *Endothiodon* subsequent to Latimer et al.’s paper^55^ has followed their convention of a single row of upper teeth and lower teeth arranged in Zahnreihen^10,11,46,56^. However, the Zahnreihen model of non-mammalian tooth replacement is not supported by developmental research^57^, so its relevance to the multiple tooth rows of *Endothiodon* is questionable.

Aside from studies on *Endothiodon*, little in-depth work has been carried out on patterns of tooth replacement in dicynodonts. Hopson^58^ suggested alternating waves of replacement teeth in the mandible of a small toothed dicynodont (identified in that paper as *Emydops* but more likely *Pristerodon*^45^), and Barry^52^ proposed an alternate pattern of replacement in different specimens of *Pristerodon*. Pathological cases of extra tusks have also been discussed^32^. However, the full diversity of replacement patterns in dicynodonts has never been considered in detail, nor how variation in replacement patterns and rates may have contributed to the evolution of the different arrangements of teeth found among dicynodonts. Given recent advances in non-destructive imaging techniques that reveal a wealth of information on internal structures of fossils^31,59–66^, along with new research on tooth attachment and replacement in other synapsids and early amniotes^6,7,67–72^, a reconsideration of tooth replacement and emplacement (i.e., the retention of several generations of teeth without replacing older teeth) in *Endothiodon* and other dicynodonts is timely. Here, we combine the first histological analysis of *Endothiodon* teeth with a computed tomographic survey of anomodonts with ‘postcanine’ teeth to describe tooth replacement in anomodonts and construct a comprehensive model of multiple tooth row evolution and development in *E. bathystoma*.

### Institutional abbreviations

BPI, Evolutionary Studies Institute, Johannesburg, South Africa; CGP, Council for Geosciences, Pretoria, South Africa; FMNH, Field Museum of Natural History, Chicago, United States of America; ML, Museu da Lourinhã, Lourinhã, Portugal; NHCC, National Heritage Conservation Commission, Lusaka, Zambia; NHMUK, Natural History Museum, London, United Kingdom; NMQR, National Museum, Bloemfontein, South Africa; NMT, National Museum of Tanzania, Dar es Salaam, Tanzania; PIN, Borissiak Paleontological Institute, Moscow, Russia; PPM, Projecto PaleoMoz, Museu Nacional de Geologia, Maputo, Mozambique. SAM, Iziko Museums of South Africa, Cape Town, South Africa; USNM, National Museum of Natural History (Smithsonian Institution), Washington D.C., United States of America.

## Materials and methods

### Materials examined

We histologically sampled ten *Endothiodon* specimens from the K5 Formation of the Metangula Graben, Mozambique^73,74^, three of which were maxillae (PPM2014-15, PPM2014-11, PPM2014-70) and the rest mandibles (PPM2014-23, PPM2014-15, PPM2014-40, PPM2014-64-17A, PPM2014-70D, PPM2014-70d, PPM2014-70-3). The mandibles PPM2014-23, PPM2014-70-6D, PPM2014-70-6d, PPM2014-70-3 have two to three tooth rows, whereas the maxillae PPM2014-15, PPM2014-40, PPM2014-64-17A only have one tooth row. Specimens PPM2014-11 and PPM2014-15 have been confidently ascribed to *Endothiodon tolani*, whereas the remaining specimens have been ascribed to *Endothiodon* sp.^10^. *Endothiodon tolani* can be readily differentiated by the presence of a caniniform tusk, which is absent in *E. bathystoma*^10,46^. Importantly, there is no stratigraphic separation between the *E. bathystoma* and *E. tolani* specimens^10^, nor between single- versus multiple-tooth-rowed specimens from the Metangula Graben (RA personal observation). More mandibles than maxillae were chosen because of their greater availability and because there is more variability in the number of mandibular tooth rows in the Mozambican specimens (no maxillae with multiple tooth-rows have been found in Mozambique).

We also compared µ-CT scans of the following anomodonts from South Africa (Karoo Basin), Tanzania (Ruhuhu Basin), Mozambique (Metangula Graben), and Zambia (Luangwa and Mid-Zambezi basins): *Patranomodon nyaphulii* maxillae (NMQR 3000), *Eodicynodon oosthuizeni* maxillae (BPI/1/6230 and NMQR 2978) and mandible (BPI/1/6230), *Pristerodon mackayi* mandible (NHCC LB190) and maxillae (NHCC LB231), *Brachyprosopus broomi* mandible and maxillae (FMNH UR 2513), *Compsodon helmoedi* mandible and maxillae (NHCC LB211), *Niassodon mfumukasi* mandible and maxillae (ML 1620), *Abajudon kaayai* mandible and maxillae (NHCC LB314), *Endothiodon tolani* mandible and maxillae with single tooth rows (NHCC LB684), *Endothiodon bathystoma* mandible with partial multiple tooth rows (NHCC LB816), *Endothiodon bathystoma* mandible with multiple tooth rows (NHCC LB11), and *Endothiodon bathystoma* maxilla with multiple tooth rows (NMT RB23).

BPI/1/6230 (*Eodicynodon*) and NHCC LB211 (*Compsodon*) are considerably smaller than other members of their species and were at first suspected to represent juveniles. However, both specimens lack osteological features that would be expected in an immature individual. The braincase is well ossified in both specimens, a feature that appears before adulthood in both mammals and sauropsids^75–79^. The proportion between the orbit and skull is indistinguishable from that of larger specimens of each species^80^, and neither specimen exhibits any open within-bone sutures that could indicate a juvenile status^79,81–86^. Finally, BP/1/6230 possesses a double canine. Although this feature can relate to ontogeny or phylogeny in some taxa^87–90^, this condition in dicynodonts is considered to be pathological, as dicynodonts have open-rooted tusks^32,90–92^. Therefore, we consider both of these specimens to represent small, but skeletally mature individuals.

### Histological sectioning

Histological specimens were impregnated with Struers^®^ epoxy resin that set for 24 h at room temperature to guarantee the integrity of the specimens during the cutting and polishing phases of the process. The first cut of each specimen was performed in a cutting machine at low rotational speeds. The cut side of the specimen was polished with 600 µm silicon carbide to ensure a smooth and uniform surface. The polished surface of the specimen was then glued to a slide with Struers^®^ epoxy, and the specimen was cut and polished using 600 µm and 320 µm silicon carbide until adequate transparency was achieved (~ 50–300 µm).

Observations were made with a Nikon^®^ eclipse C*i* POL microscope and photos were taken using the coupled camera. Image post-processing was done using Faststone Image Viewer 7.4, and Photo Stitcher 2.1 was used to combine overlapping photos to produce a panoramic image.

### µ-CT scanning

Specimens were µ-CT scanned at the following facilities:

- Evolutionary Studies Institute, University of the Witwatersrand (Nikon Metrology XTH 225/320 LC): NMQR 3000 (*Patranomodon*, voxel size 35.6 µm, voltage 70 kV, current 80 µA), BP/1/6230 (*Eodicynodon oosthuizeni*, voxel size 64.6 µm, voltage 100 kV, current 140 µA), NMQR 2978 (*Eodicynodon oosthuizeni*, voxel size 73.9 µm, voltage 100 kV, current 140 µA).
- University of Chicago PaleoCT Facility (GE Phoenix V|tome|x S240): NHCC LB190 (*Pristerodon,* voxel size 24.3 µm, voltage 110 kV, current 220 µA), NHCC LB231 (*Pristerodon,* voxel size 22.9 µm, voltage 100 kV, current 220 µA), NHCC LB211 (*Compsodon*, voxel size 40.5 µm, voltage 190 kV, current 200 µA), NHCC LB314 (*Abajudon*; mandible: voxel size 36.6 µm, voltage 200 kV, current 300 µA; maxillae: voxel size 80.1 µm, voltage 120 kV, current 660 µA), NHCC LB684 (*E. tolani*, voxel size 116.7 µm, voltage 215 kV, current 200 µA).
- Baker Hughes GE Inspection Technologies Facility (GE Phoenix V|tome|x M300): FMNH UR 2513 (*Brachyprosopus*, voxel size 15 µm, voltage 270 kV, current 65 µA).
- European Synchrotron Radiation Facility, Grenoble, France (ID19): ML1620 (*Niassodon*, voxel size 27.85 µm, detected total integrated energy at 97.8 keV).
- University of Washington Computed Tomography Facility (NSI X5000): NHCC LB816 (*E. bathystoma*, voxel size 61 µm, voltage 220 kV, current 470 µA), NHCC LB11 (*E. bathystoma*, voxel size 126 µm, voltage 220 kV, current 470µA).
- Friday Harbor Laboratories (Skyscan 1173): NMT RB23 (*E. bathystoma*, voxel size 71.4 µm, voltage 130 kV, current 61 µA).

Teeth were segmented using Avizo V9.2.0 (© FEI VSG, Hillsboro OR, USA). This program was also used to visualize sections through most specimens, although specimens of *Patranomodon* and *Eodicynodon* were visualized using Dragonfly 2020.1.0.11657 (ORS Inc., Montreal, Canada).

## Results

We adopt the terminology presented by Hendrickx et al.^93^ for our descriptions of tooth and tooth row anatomy.

### Histological sections of *Endothiodon*

#### Overview

The number of tooth rows is variable and unrelated to specimen size or stratigraphic position. New teeth were added linguodistally, and in multi-rowed individuals the functional teeth were not replaced upon eruption of new teeth. Replacement teeth are not seen in single-rowed individuals, possibly reflecting differences in rates of new tooth formation between single- and multi-rowed individuals. The specimens have thecodont tooth implantation and a matrix-infilled space around each tooth that we interpret as once housing a periodontal ligament. This space is surrounded by a layer of alveolar bone, to which the ligament would have attached in life.

#### Dentary

##### Dentition

An individual dentary tooth is a straight rod (i.e., there is no basal constriction of the crown) with a falciform crown ornamented with distal denticles when not worn by tooth-to-tooth contact (see Latimer et al.^55^). Teeth are subcircular in cross-section. There are typically about 7–12 denticles on each tooth, and the denticle apices point distally or slightly basally. Denticle size is uniform apicobasally, although the most apical denticle tends to be slightly smaller in size. The denticle operculum is pointed. The interdenticular slit engulfs the entirety of the denticle; therefore, there is no interdenticular diaphysis. There are also no interdenticular sulci.

Tooth diameter and morphology are consistent along the jaw. The number of tooth rows is not related to size or ontogenetic stage, as various specimens from Mozambique and South Africa have been found in the same horizon and with subequal sizes while possessing single or multiple tooth-rows (RA and AD personal observation). A newer tooth was always emplaced on the linguodistal side of the older tooth, but in some cases, it could lie directly underneath the older tooth (PPN2014-70-3). However, the labialmost teeth are not lost; they are continuously accreted lingually.

##### Dental histology and implantation

**Figure 2:**
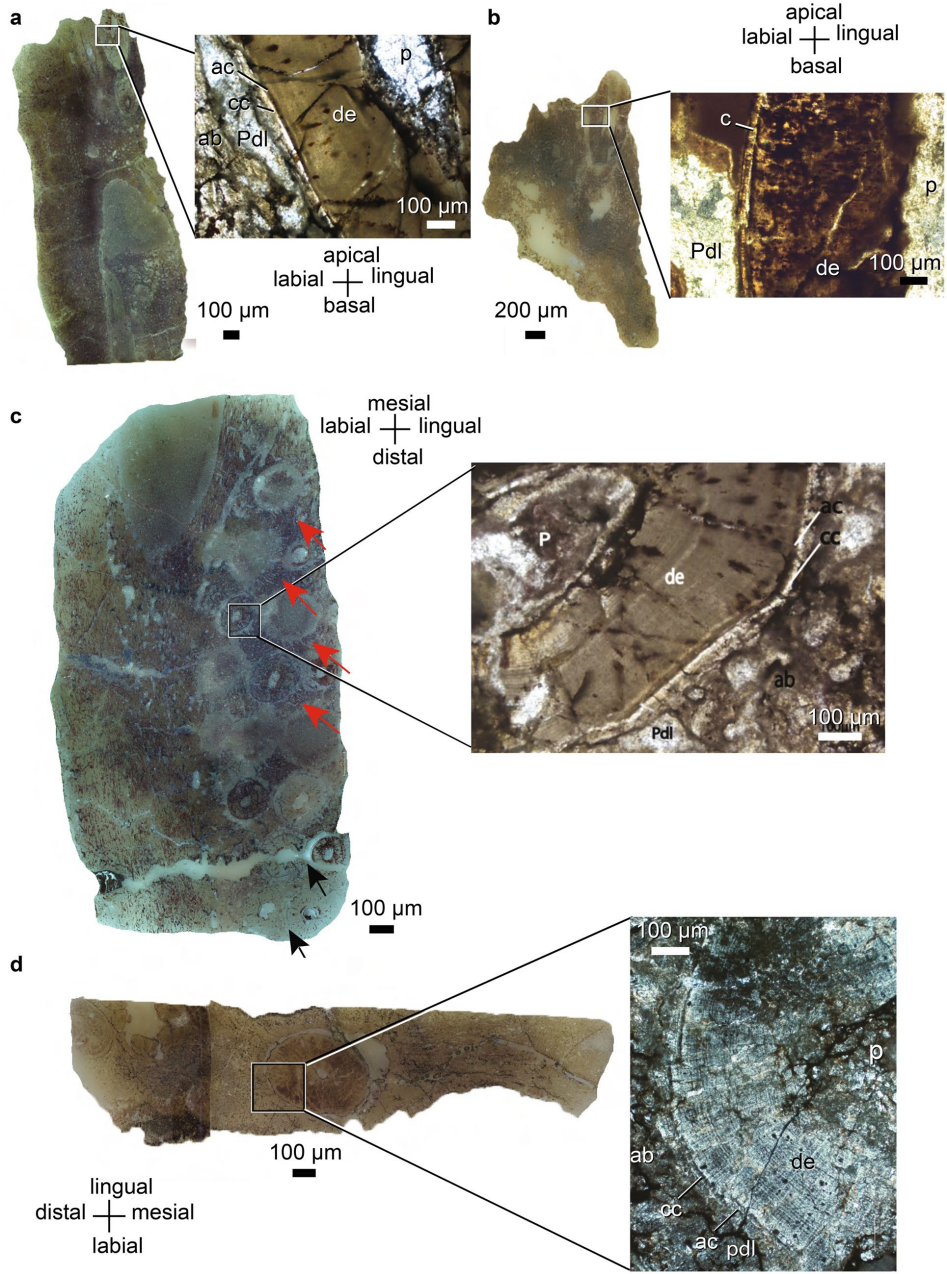
Dentary histological sections of *Endothiodon* sp. specimens from the K5 Formation, Metangula Graben, Mozambique. (**a**) Coronal section of the three-toothrow dentary of PPN2014-70-3. (**b**) Coronal section of the single-toothrow dentary of PPN2014-64-17A. (**c**) Horizontal section of the three-toothrow dentary of PPN2014-70-3. The red arrows depict the trails left by the tooth migration and the black arrows depict the cross-cutting patterns between tooth generations, indicating that there is a labial to lingual migration of the teeth through development. (**d**) Horizontal section of the single-toothrow dentary of PPN2014-15. *ab* alveolar bone, *ac* acellular cementum, *c* cementum, *cc* cellular cementum, *de* dentine, *p* pulp cavity, *pdl* periodontal ligament space.

**Figure 3:**
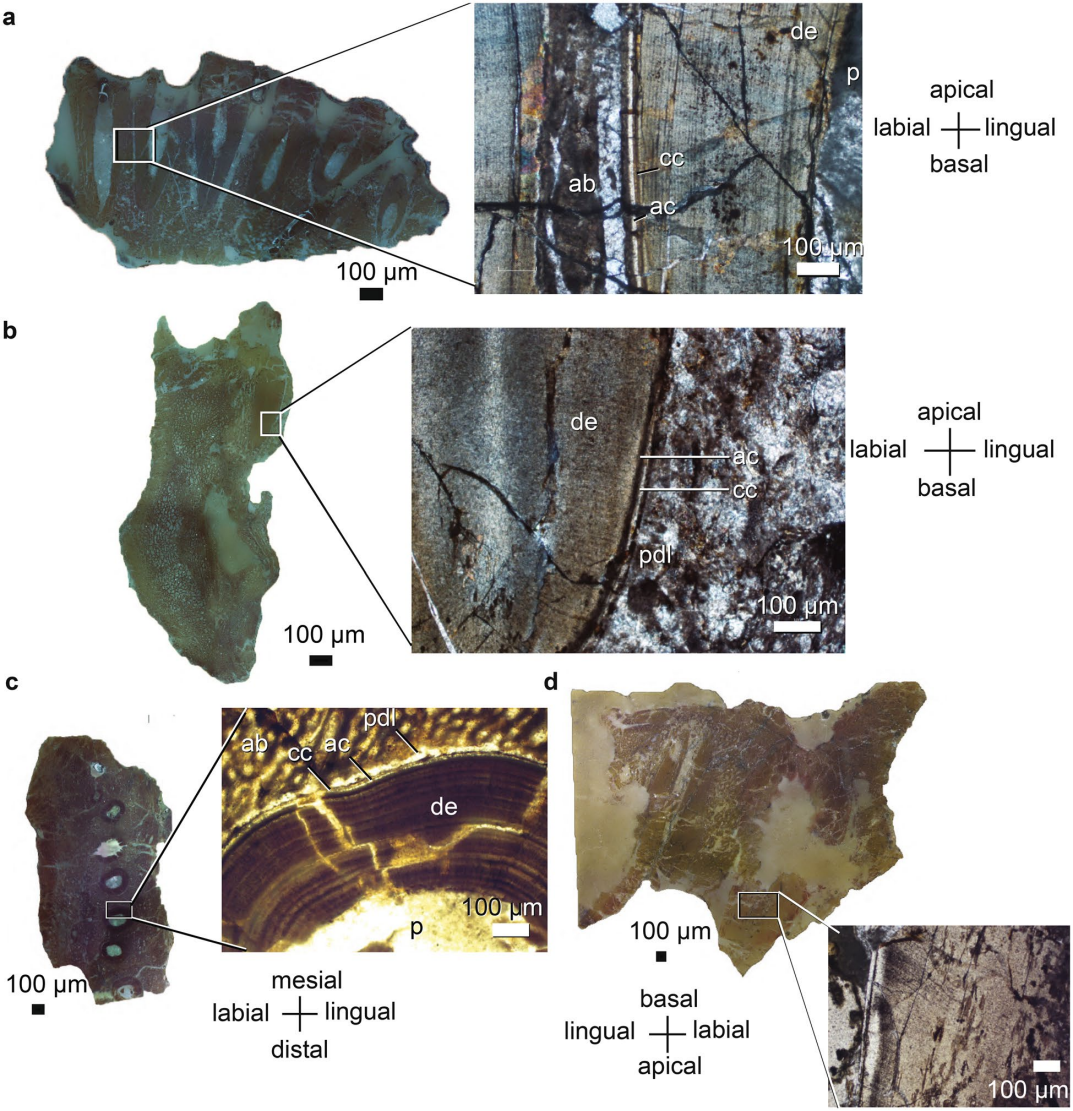
Maxillary histological sections of *Endothiodon tolani* specimens from the K5 Formation, Metangula Graben, Mozambique. (**a**) Sagittal section of the PPN2014-11 maxilla. (**b**) Coronal section of the PPN2014-15 maxilla. (**c**) Horizontal section of the PPN2014-11 maxilla. (**d**) Coronal section of the PPN2014-15 maxilla. *ab* alveolar bone, *ac* acellular cementum, *cc* cellular cementum, *de* dentine, *p* pulp cavity, *pdl* periodontal ligament space.

*Endothiodon* teeth have a central pulp cavity surrounded by dentine and are coated in two thin layers of cellular and acellular cementum. There is no enamel layer on the sampled crowns. However, it is necessary to section an unworn, denticled tooth to ascertain the absence of enamel, as it is expected that dentine is typically the main constituent of denticles, and that the enamel layer would likely be quite thin^94,95^. The type of implantation is thecodont, meaning the teeth are deeply and symmetrically implanted within U-shaped alveoli^70^. The teeth are attached to the socket via a gomphosis; they are not directly ankylosed (fused) to the mandibular bone, but instead were suspended by periodontal ligaments within each alveolus during life. The soft tissue of the periodontal ligament is not preserved, but a space persists between the tooth root surface and the surrounding alveolar bone, that would have housed the ligament in life (Figs. 2, 3). The alveolar bone surrounding the tooth is well vascularized, with small vascular channels near the tooth that become progressively larger away from the tooth. In some cases, the vascular channels are oriented radially around the tooth (PPN2014-70-3), but usually there is no particular orientation in the regions closer to the jawbone. The vascular channels have particular orientations in areas away from the teeth, such as near the Meckelian canal or the lateral wall of the mandible. No reversal lines between the layers of alveolar bone and the bone of the jaw were visible.

The pulp cavity contained the vital components of the tooth in life. The extension and development of the pulp cavity can best be seen in coronal or sagittal views, but its ellipsoidal cross-section can best be seen in horizontal cross-section (Fig. 2a–c, respectively). The major axis of the horizontal cross-section is oriented roughly labiolingually. The pulp cavities typically have a teardrop or ogive shape, depending on whether they are cut offset or through the median section of the tooth, respectively. The pulp cavity averages ~ 500 µm in thickness in horizontal view, but it can range from 270 to 1200 µm (Table 1).

**Table 1:** Thicknesses of tooth layers seen in histological sections.

Specimen	Taxon	Element	Section	Pulp cavity maximal width (µm)	Dentine thickness (µm)	Acellular cementum thickness (µm)	Cellular cementum thickness (µm)	Periodontal space thickness (µm)
PPN2014-70-6D	*Endothiodon* sp.	Mandible	Coronal	800	400	3.4	6.8	360 to 980 (between the teeth)
			Horizontal	500 (mesially) to 1200 (distally)	500	9	12	350
PPN2014-70-6d	*Endothiodon* sp.	Mandible	Coronal	450	–	–	15	–
			Horizontal	450	–	–	45	20
PPN2014-70-3	*Endothiodon* sp.	Mandible	Coronal	300 on older teeth, 200 in the new tooth	420	11.8	14.7	20–100
			Horizontal	500 (mesially) to 300 (distally)	350	10	25	5–200 in the single tooth row region, 50 in the multiple tooth row region
			Sagittal	200	250	10	25	120
PPN2014-15	*Endothiodon tolani*	Mandible	Horizontal	270	–	15	35	90
			Coronal	–	300	15	15	130
		Maxilla (‘postcanines’)	Coronal	–	–	18	12	295
			Horizontal	–	400	–	–	80
			Sagittal	–	400	–	–	–
		Maxilla (tusk)	Coronal	–	650	–	–	180
PPN2014-64-17A	*Endothiodon* sp.	Mandible	Coronal	700	400	–	–	200
			Horizontal	500	500	–	–	100
PPN2014-40	*Endothiodon* sp.	Mandible	Coronal	350	150	–	–	100
PPN2014-11	*Endothiodon tolani*	Mandible	Horizontal	400	360	12	10	8
			Sagittal	400	300	16	13	0
		Maxilla (‘postcanines’)	Sagittal	530	300	13	16	–
PPN2015-70	*Endothiodon* sp.	Mandible	Horizontal	400	300	20	18	95
PPN2014-23	*Endothiodon* sp.	Mandible	Horizontal	400	300	20	18	95

Dentine composes nearly the entirety of the tooth hard parts in *Endothiodon*. In horizontal cross-section it is often possible to observe radially oriented dentine tubules (Figs. 2b–d, 3a,d), and perpendicular to them run what may be von Ebner lines (Figs. 2c,d, 3a,c,d). The dentine layer ranges from 300 to 500 µm in horizontal section (Table 1).

As in the teeth of mammals and other fossil synapsids^69^, layers of cementum coat the roots of the teeth. The acellular cementum layer is closer to the dentine than the cellular cementum layer is. The cellular cementum layer is usually dark brown whereas the acellular cementum layer is highly transparent. The cellular cementum layer is generally thicker than the acellular cementum layer, the former ranging from 6.8 to 35 µm, whereas the latter ranges from 3.4 to 20 µm (Table 1). The acellular and cellular cementum layers are not distinguishable in PPN2014-64-17A and PPN2014-40 in horizontal or coronal section.

We observed in the distalmost teeth of PPN2014-70-3 that attachment tissues from younger (i.e., more lingual) teeth cross-cut older (i.e., more labial) teeth. Furthermore, it is possible to observe in several instances along the jaw a trail of alveolar bone with bone trabeculae linearly oriented in a labiomesial to linguodistal direction.

The matrix-filled space between the alveolar bone and the tooth itself likely corresponded to the periodontal ligament when the animal was alive. The thickness of the periodontal space averages ~ 160 µm but there is a high degree of variation in these measurements, ranging from an apparent absence of space for the periodontal ligament in places where neighboring tooth attachment tissues cross-cut one another (Figs. 2c, 3a)(PPN2014-11) to 980 µm between teeth in PPN2014-70-6D (Table 1). There seems to be no correlation between the orientation of the section and the thickness of the periodontal ligament. Rather, the differences seem to be related to the individual teeth measured and likely reflect the spacing between teeth. Newly emplaced teeth deposit a layer of alveolar bone around their periodontal space, and this can invade the periodontal space of neighboring teeth if they are close enough (Figs. 2c, 3a). There is no difference between *E. bathystoma* and *E. tolani* in the composition or proportions of dental tissues and implantation of the teeth (see Figs. 2, 3).

#### Maxilla

##### Dentition

Apart from the presence of the caniniform tusk, there is no difference in the maxillary teeth of *E. tolani* and *E. bathystoma.* The specimens analyzed here possess a single maxillary tooth row. In horizontal section, the maxillary teeth of *E. tolani* (PPN2014-11) are arranged in a relatively straight row, but in *E.* sp. (PPN2014-70) there is some labiolingual offset between the teeth. The maxillary tooth rows are parallel to the borders of the maxilla but are slightly oblique relative to the sagittal plane. The tooth roots are ellipsoidal in cross-section with the major axis oriented labiolingually and sometimes with the lingual side oriented somewhat mesially. A small, newer tooth is located mesiolabially relative to the main maxillary tooth row in PPN2014-11 (Fig. 3c).

In sagittal section, the *E. tolani* maxillary teeth seem to progressively tilt distally, but the replacement teeth are not so obliquely oriented (see Fig. 3a). A similar condition is observed in PPN2014-15 and the CT scan of *Abajudon* (Fig. 9b). None of the specimens described here preserve complete teeth, so wear facets are not visible, but labial and lingual facets have been described by Latimer et al.^55^. The caniniform tusk lies labial to the maxillary tooth row. The tusk is not as developed as in most dicynodont taxa, yet there is a broadly developed alveolus (e.g., PPN2014-11, PPN2014-15).

##### Dental histology and implantation

The maxillary tooth implantation and histology are similar to those of the dentary teeth. The implantation is thecodont, and the teeth were fixed via a gomphosis. There is a vascularized alveolar bone layer to which the periodontal ligament would have attached. The tooth itself is formed primarily of dentine with a layer of acellular cementum surrounded by a thin layer of cellular cementum (see Table 1 for thicknesses). The pulp cavity is usually filled with sediment.

### CT scans

In addition to understanding the mechanics by which multiple tooth rows developed in *E. bathystoma*, we also aimed to characterize the phylogenetic context from which this distinctive dentition evolved and reveal features that may have contributed to its appearance. We used computed tomography to visualize tooth replacement and emplacement in an array of dicynodonts with ‘postcanines’. Our survey allowed us to document variation in ‘postcanine’ tooth row morphology and replacement dynamics among dicynodonts.

#### Tooth attachment

Most extant reptiles have their functional teeth fused to the jaw bone (ankylosis), whereas mammals, crocodilians, and a growing list of extinct amniotes have a soft-tissue attachment of teeth to bone (gomphosis), mediated by the periodontal ligament^96,97^. Recent work has shown that all teeth have a gomphosis early in development, meaning that the dichotomy between a gomphosis and ankylosis actually reflects two ends of an ontogenetic spectrum of tooth attachment tissue mineralization^7^.

**Figure 4:**
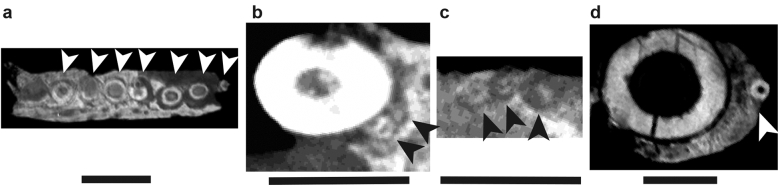
µCT scans of mandibular ‘postcanine’ teeth in horizontal section showing evidence for a gomphosis at the base of Dicynodontia. (**a**) *Patranomodon nyaphulii* maxillary teeth (NMQR3000, mirrored), (**b**) *Eodicynodon oosthuizeni* tusk and maxillary ‘postcanines’ (BPI/1/6230), (**c**) *Eodicynodon oosthuizeni* mandibular teeth (BPI/1/6230, mirrored), (**d**) *Eodicynodon oosthuizeni* tusk and maxillary tooth (NMQR 2978, mirrored). Scale bar 5 mm. Anterior is to the left, lingual is up. Arrows indicate ‘postcanine’ teeth.

**Figure 5:**
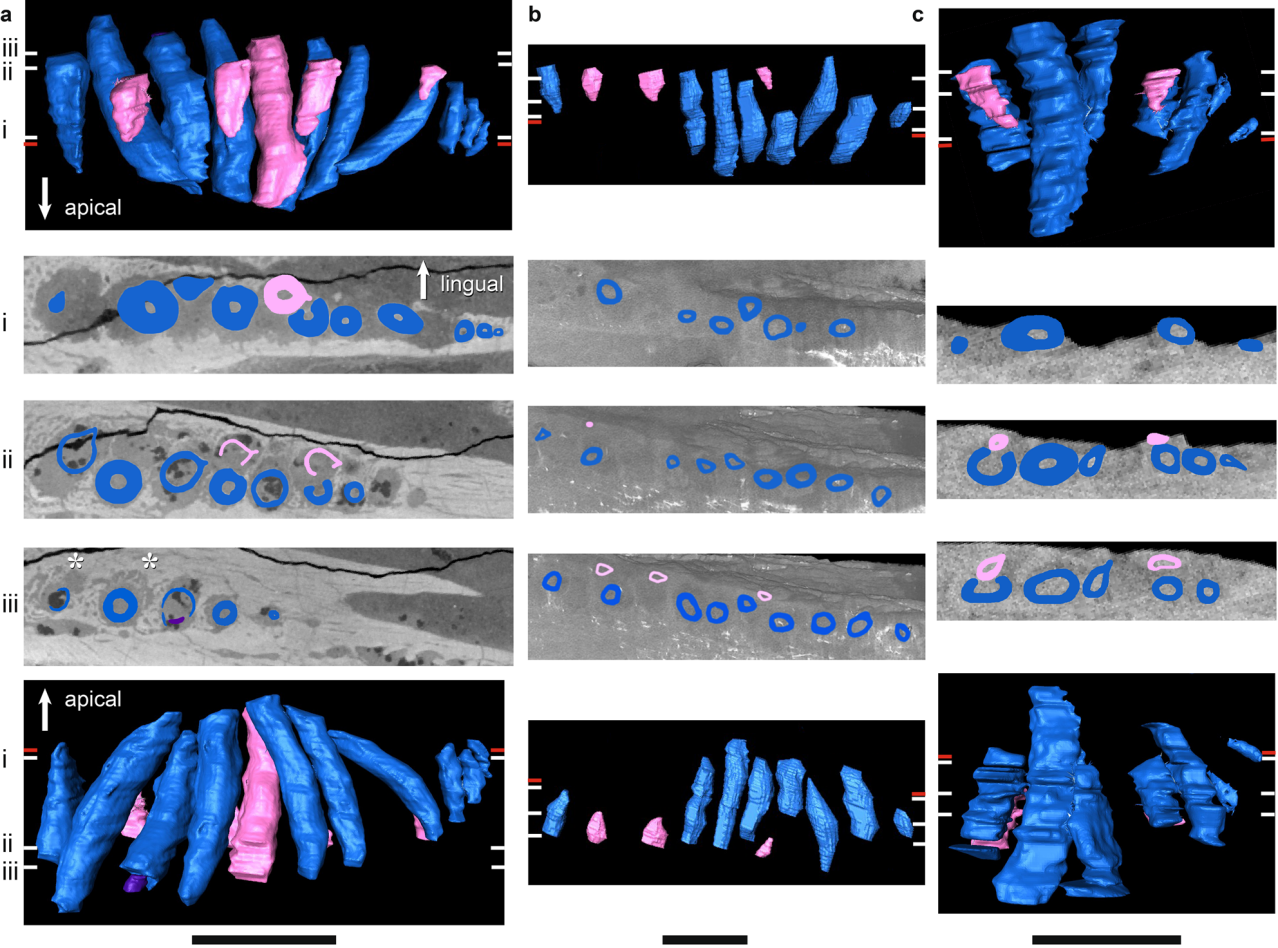
µCT scans of basal dicynodont mandibular ‘postcanine’ teeth in horizontal section (middle three rows), lingual view (top row), and labial view (bottom row). (**a**) *Pristerodon mackayi* NHCC LB190, (**b**) *Brachyprosopus broomi* FMNH UR 2513, (**c**) *Compsodon helmoedi* NHCC LB211. Horizontal sections were taken at numbered positions indicated on the labial and lingual views. The level of the gumline is indicated by a red tick mark on labial and lingual views. Replacement teeth (i.e., those that are eroding a more labial tooth) are highlighted in light pink, functional teeth in medium blue, and tooth remnants in dark purple. Anterior is to the left. Scale bars 5 mm. Asterisks indicate visible alveolar bone trails.

**Figure 6:**
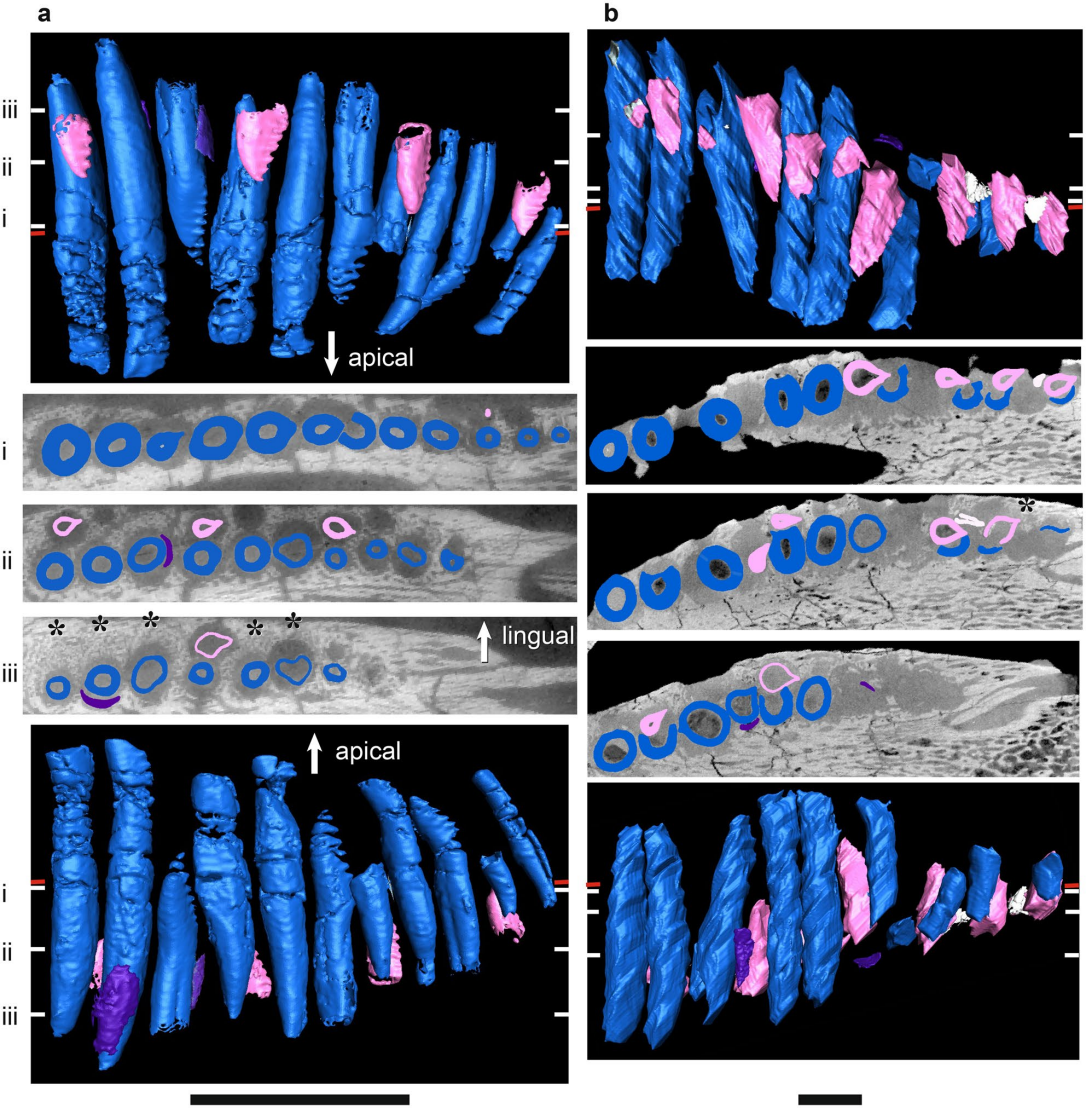
µCT scans of basal endothiodont mandibular ‘postcanine’ teeth in horizontal section (middle three rows), lingual view (top row), and labial view (bottom row). (**a**) *Niassodon mfumukasi* ML 1620 (mirrored), (**b**) *Abajudon kaayai* NHCC LB314 (mirrored). Horizontal sections were taken at numbered positions indicated on the labial and lingual views. The level of the gumline is indicated by a red tick mark on labial and lingual views. First-generation replacement teeth are highlighted in light pink, second-generation replacement teeth in white, functional teeth in medium blue, and tooth remnants in dark purple. Anterior is to the left. Scale bars 5 mm. Asterisks indicate visible alveolar bone trails.

**Figure 7:**
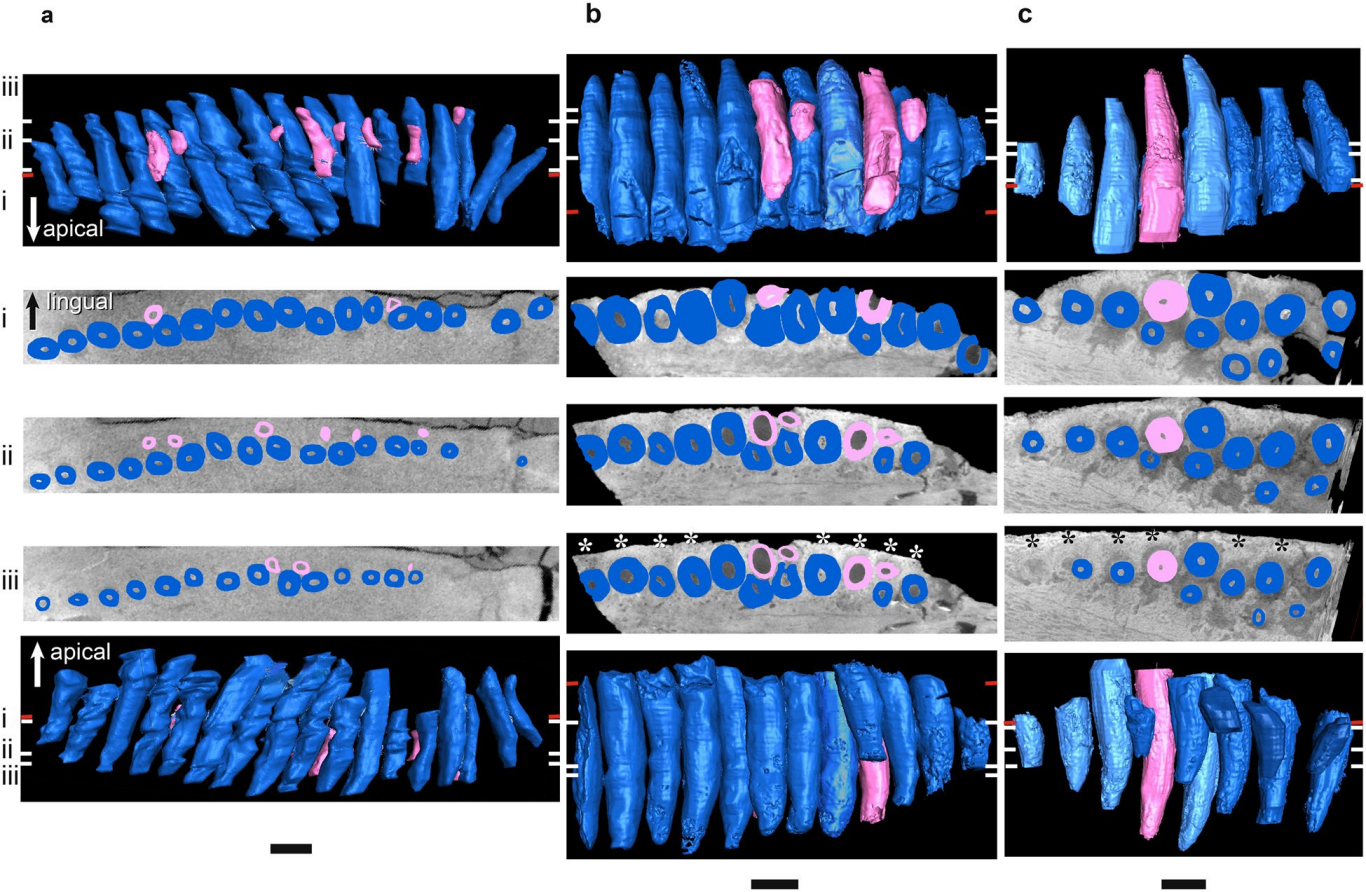
µCT scans of *Endothiodon* mandibular ‘postcanine’ teeth in horizontal section (middle three rows), lingual view (top row), and labial view (bottom row). (**a**) *Endothiodon tolani* NHCC LB684 (mirrored), (**b**) *Endothiodon bathystoma* NHCC LB816 with partial multiple rows (mirrored), (**c**) *Endothiodon bathystoma* NHCC LB11 with multiple rows (mirrored). Horizontal sections were taken at numbered positions indicated on the labial and lingual views. The level of the gumline is indicated by a red tick mark on labial and lingual views. Replacement teeth are highlighted in light pink and functional teeth in medium blue. Anterior is to the left. Scale bars 5 mm. Asterisks indicate visible alveolar bone trails.

**Figure 8:**
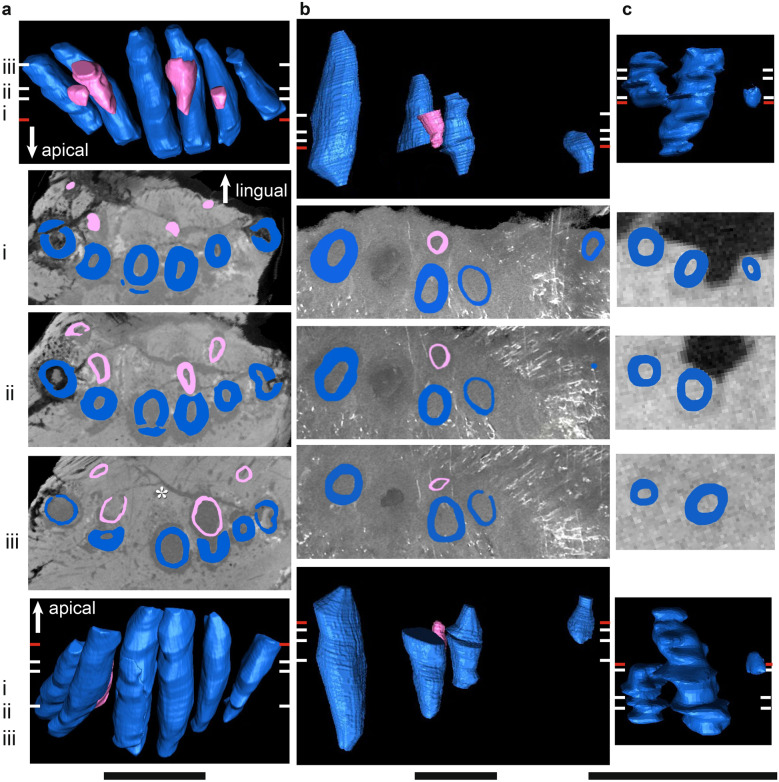
µCT scans of basal dicynodont maxillary ‘postcanine’ teeth in horizontal section (middle three rows), lingual view (top row), and labial view (bottom row). (**a**) *Pristerodon mackayi* NHCC LB231 (mirrored), (**b**) *Brachyprosopus broomi* FMNH UR 2513, (**c**) *Compsodon helmoedi* NHCC LB211 (mirrored). Horizontal sections were taken at numbered positions indicated on the labial and lingual views. The level of the gumline is indicated by a red tick mark on labial and lingual views. Replacement teeth are highlighted in light pink and functional teeth in medium blue. Anterior is to the left. Scale bars 5 mm. Asterisks indicate visible alveolar bone trails.

**Figure 9:**
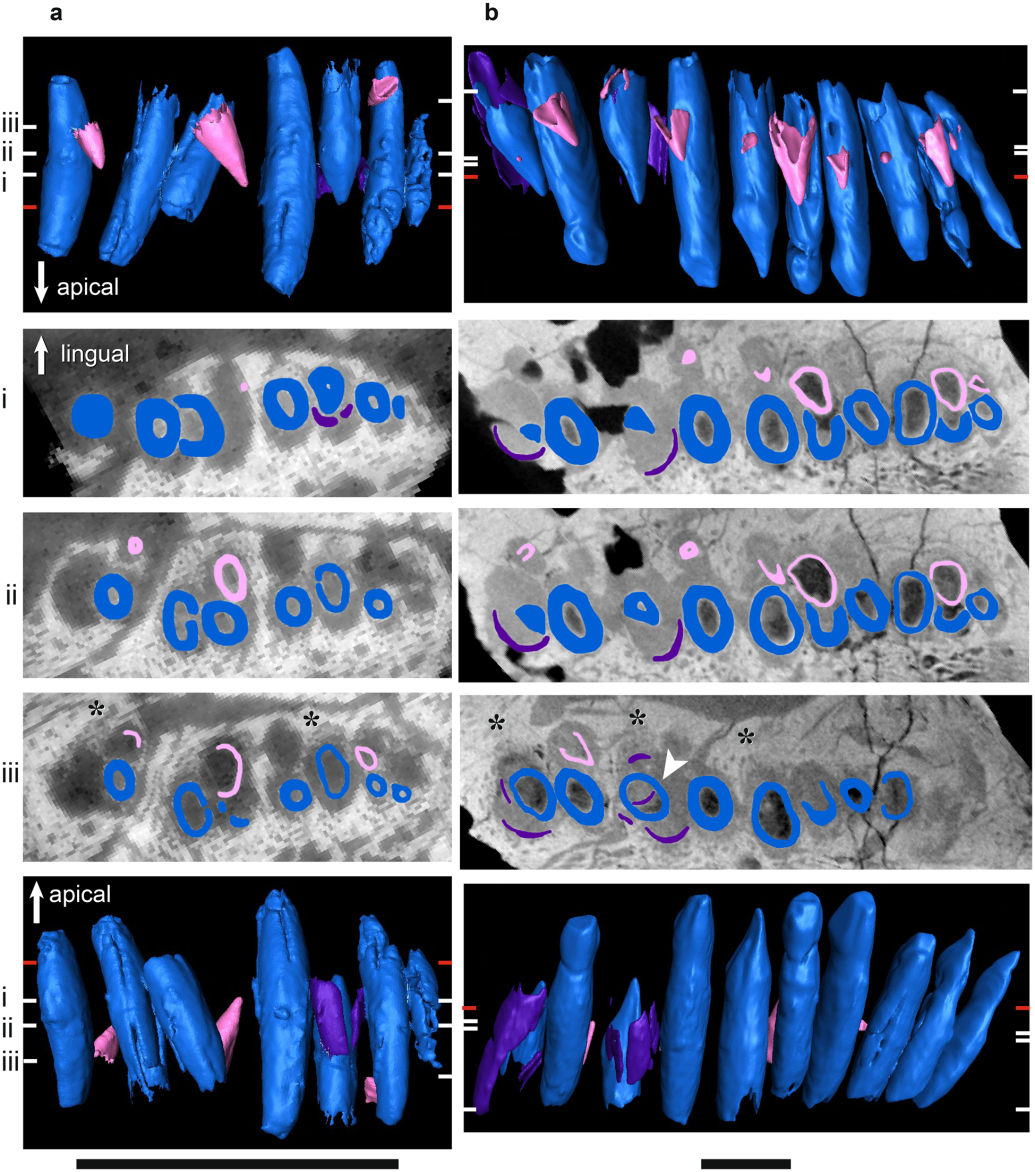
µCT scans of basal endothiodont maxillary ‘postcanine’ teeth in horizontal section (middle three rows), lingual view (top row), and labial view (bottom row). (**a**) *Niassodon mfumukasi* ML 1620, (**b**) *Abajudon kaayai* NHCC LB314 (mirrored, white arrow pointing to remnant included in tooth pulp cavity). Horizontal sections were taken at numbered positions indicated on the labial and lingual views. The level of the gumline is indicated by a red tick mark on labial and lingual views. Replacement teeth are highlighted in light pink, functional teeth in medium blue, and tooth remnants in dark purple. Anterior is to the left. Scale bars 5 mm. Asterisks indicate visible alveolar bone trails.

**Figure 10:**
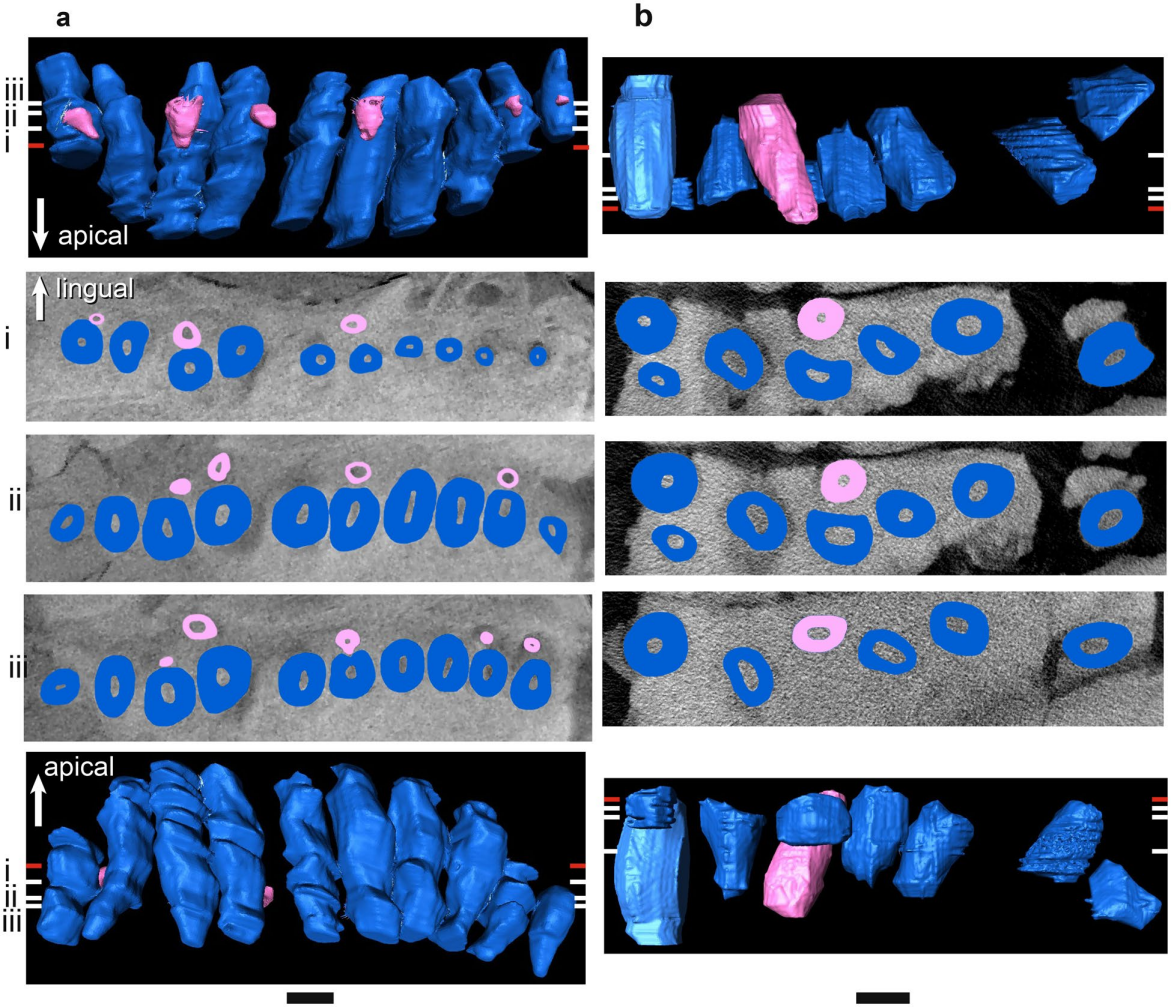
µCT scans of *Endothiodon* maxillary ‘postcanine’ teeth in horizontal section (middle three rows), lingual view (top row), and labial view (bottom row). (**a**) *Endothiodon tolani* NHCC LB684 (mirrored), (**b**) *Endothiodon bathystoma* NMT RB23 with multiple rows. Horizontal sections were taken at numbered positions indicated on the labial and lingual views. The level of the gumline is indicated by a red tick mark on labial and lingual views. Replacement teeth are highlighted in light pink and functional teeth in medium blue. Anterior is to the left. Scale bars 5 mm.

A gomphosis has been documented in several non-mammalian therapsids^69,72^, including the dicynodonts *Abajudon*^47^ (‘postcanines’) and *Lystrosaurus* (tusks)^98^. In the anomodonts examined here, the CT scans show spaces around the ‘postcanine’ teeth that were originally occupied by the periodontal ligament and were infilled by matrix after death (Figs. 4, 5, 6, 7, 8, 9, 10)^69,89^. This is also the case in the histological sections of *Endothiodon* sp. and *Endothiodon tolani* (see above, Figs. 2, 3). These spaces are found around developing teeth as well as fully mature functional teeth, suggesting that nearly all the anomodonts examined here possessed a permanent gomphosis around their ‘postcanines’. It is unclear in the scans of *Compsodon* and *E. tolani* whether or not there is a periodontal space around the teeth, although histological sectioning confirms its presence in at least some specimens of the latter. Likewise, Angielczyk and Kammerer^99^ reported empty ‘postcanine’ alveoli in other specimens of *Compsodon* that are suggestive of the presence of a periodontal ligament that decayed following death, allowing the teeth to fall out.

The periodontal space is fairly large in most of the anomodonts in our sample, ranging from 86 (*E. bathystoma* NHCC LB816) to 457 µm (*Abajudon*, NHCC LB314) in width. Many of the mandibles in our sample had periodontal spaces that are roughly continuous between adjacent tooth positions. In a few specimens (*Pristerodon* NHCC LB190, *Abajudon* NHCC LB314, and *E. bathystoma* NHCC LB11; Figs. 5a, 6b, 7c), adjacent periodontal spaces are so closely set that they form a trough along the entire tooth row. The only CT-scanned specimen that clearly shows a thin periodontal space is NHCC LB816 (*E. bathystoma*). In general, the maxillae had moderately-sized periodontal spaces, although the spaces are smaller than in the mandibles and the spaces are not continuous between tooth positions in the maxillae of our scans. However, other specimens of *Pristerodon* in the literature seem to have a continuous periodontal space between maxillary tooth positions^39^ (Fig. 1), and the periodontal spaces are nearly continuous between tooth positions in *Abajudon*. The only maxilla that seems to have a small periodontal space is NMT RB23 (*E. bathystoma*), although the low contrast of the scan makes this difficult to confirm. Some specimens show a thinner gomphosis surrounding the more mature teeth (Figs. 5a, 6a, 8a, 9), suggesting that the typical ontogenetic transition from a gomphosis to ankylosis seen among amniotes^7^ still occurs in these taxa, but it happens at a slower pace that allows the gomphosis to be retained in fully mature teeth.

#### Tooth replacement mode

In most amniotes, replacement teeth develop lingual to the functional teeth they will eventually replace, and they develop sequentially in a wave-like pattern in alternating tooth positions that progresses mesiodistally along the length of the tooth row^3^. However, we noted some variation in the positions of replacement teeth in our endothiodont sample, and there are occasional teeth that do not follow the same replacement cycle as the rest of the tooth row in a specimen (Fig. 6a(i), 9a(i),b(i)). Among anomodonts, replacement mode seems to vary most within *Endothiodon*, with some specimens showing distolingual (Fig. 7a(iii)), lingual (Fig. 7a,b), or mesiolingual (Fig. 7c) replacement, although our Mozambican histological sample exhibits exclusively distolingual replacement (Fig. 2c).

The partially-multiple-rowed *E. bathystoma* specimen NHCC LB816 exhibits slight resorption of labial teeth by adjacent lingual teeth, as well as by some of the teeth in neighboring tooth positions (Fig. 7b). The cross-cutting relationships of these teeth indicate a typical replacement pattern in this specimen, with the lingual teeth resorbing labial teeth in alternating positions along the tooth row. However, the older teeth have avoided complete resorption by the lingual teeth. In the fully-multiple-rowed *E. bathystoma* (NHCC LB11), there is almost no resorption occurring, but the teeth are arranged at a slight angle to one another. The one tooth in this specimen that does exhibit erosion is being resorbed by a mesiolingual tooth (Fig. 7c). The more lingual tooth at this position is fully mature and erupted, so it is unlikely that it would have ever replaced the more labial tooth. In the multi-rowed mandible NMT RB23, the teeth are eroded by more lingual teeth. Similar patterns of erosion can be seen in our histological sections of *Endothiodon* (Fig. 2c).

**Figure 11:**
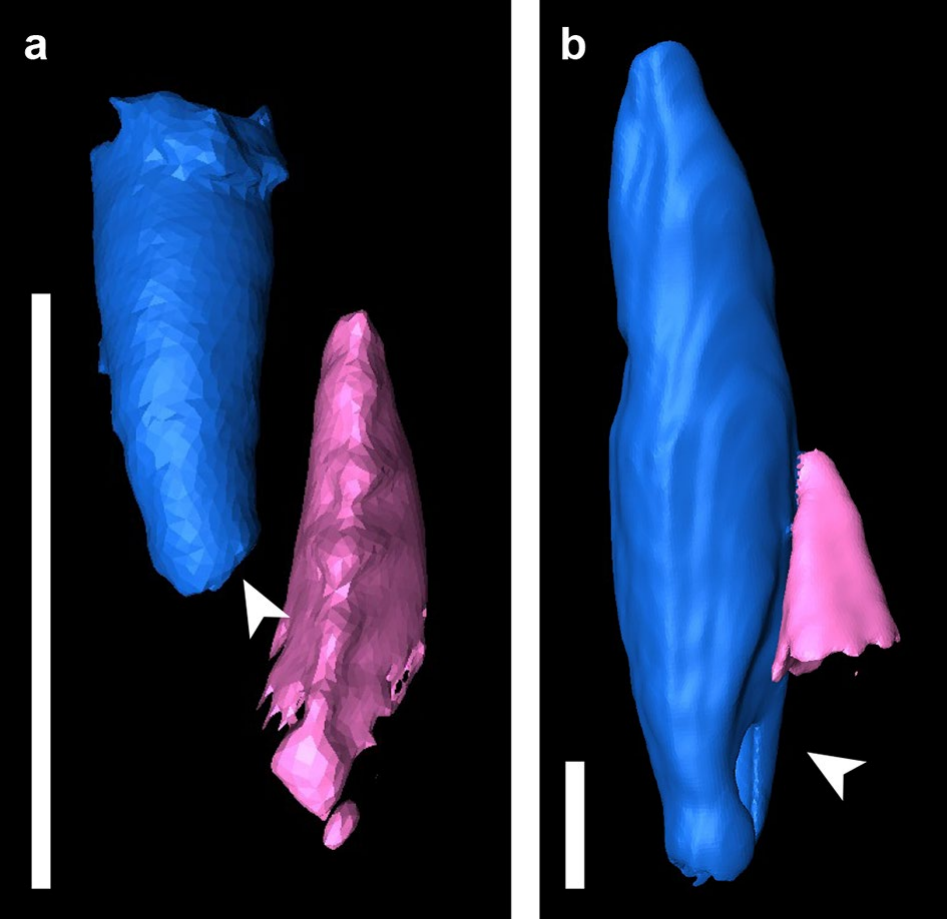
Segmented µCT scanned teeth in distal view showing the onset of erosion. (**a**) *Niassodon mfumukasi* mandibular teeth ML 1620, (**b**) *Abajudon kaayai* maxillary teeth NHCC LB314. The crown apex is up and lingual is to the right. Replacement teeth are highlighted in light pink and functional teeth in medium blue. White arrows indicate erosional surfaces on functional teeth. Scale bars 2 mm.

The replacement teeth are oriented roughly parallel to the functional teeth in the coronal plane during replacement (Fig. 11). There is some variation between taxa in the site of tooth resorption initiation. In *Compsodon* and *Niassodon*, resorption begins near the root apex of the functional tooth, but in the upper jaw of *Abajudon* and one specimen of *E. bathystoma* (NHCC LB816), resorption begins near the base of the crown (Fig. 11). In all taxa except *Abajudon,* the replacement teeth start to develop just below the alveolar margin. In *Abajudon*, the upper teeth start to develop near the root apex of the functional tooth (Fig. 9b). Replacement pits in this taxon are present at the bone surface at each replacement tooth position.

#### Proxies for tooth replacement rate

Modifications to the rate at which new teeth develop and replace older teeth can play a major role in the acquisition of multiple tooth rows^6^. In our sample, there is a general trend towards having smaller, less developed replacement teeth initiating erosion of the functional teeth in the later-diverging taxa. *Abajudon* and *Endothiodon* have teeth that are smaller and presumably less developed at the onset of resorption than is seen for the other taxa (Table 2, Figs. 7b, 9b).

**Table 2:** Heights of functional and replacement teeth at the onset of erosion during the replacement process.

Species	Specimen	Element	Functional tooth height at erosion onset (mm)	Replacement tooth height at erosion onset (mm)	Ratio of replacement: functional tooth height at erosion onset
*Pristerodon mackayi*	NHCC LB190	Mandible	7.25	2.71	0.37
*Compsodon helmoedi*	NHCC LB211	Mandible	3.88	2.57	0.66
*Niassodon mfumukasi*	ML 1620	Mandible	2.76	1.18	0.43
*Abajudon kaayai*	NHCC LB314	Mandible	15.48	2.08	0.13
		Maxilla	13.67	2.75	0.20
*Endothiodon tolani*	NHCC LB648	Maxilla	20.94	5.51	0.26
*Endothiodon bathystoma*	NHCC LB816	Mandible	16.19	3.90	0.24

Note that these measurements could only be taken on specimens that captured this stage of replacement.

In the dicynodonts sampled here, there is never more than one replacement tooth per family in individuals with single tooth rows, except for a few tooth positions in the lower jaw of *Abajudon* that have two generations of replacement teeth (Fig. 6b). In the lower jaw of NHCC LB11 (*E. bathystoma)*, there are 1–3 teeth present per tooth family, with more teeth per family at the distal end of the jaw (Fig. 7c). A similar relative rate is seen in the Mozambican *Endothiodon* specimens. In the multi-rowed *E. bathystoma* mandible NHCC LB11 (Fig. 7c), the labialmost teeth are notably smaller in cross-section than the presumably younger lingual teeth. This difference indicates a substantial change in body size between tooth emplacement events, which suggests that teeth were not added frequently in this taxon^6^.

Replacement is fairly bilaterally symmetrical, with replacement teeth at similar stages present at the same tooth positions on the left and right sides of the mouth. About a third to half of the tooth positions in the whole sample have developing replacement teeth, but almost all tooth positions have replacement teeth in *Abajudon* (Figs. 6b, 9b). In most taxa, the fraction of tooth positions with replacement teeth is fairly consistent mesiodistally along the tooth row. However, none of the *Endothiodon* mandibles show evidence of replacement teeth at the mesial end of the mandible, even though there are replacement teeth in the rest of the jaw (Fig. 7). The maxillae did have new teeth being added at the mesial end (Fig. 10).

#### Tooth and socket migration

Like all other amniotes, each dicynodont tooth root is surrounded by a discrete layer of alveolar bone^7,9,96,100^. This halo-like arrangement of highly vascularized bone around each tooth is distinct from the surrounding jaw bone, even in CT scanned images (Figs. 2, 3, 4, 5, 6, 7, 8, 9). Alveolar bone is formed anew with each developing tooth and is maintained by the cells of the periodontium until the tooth is finally shed^100–102^. In most cases, the alveolar bone surrounding a tooth is circular in cross-section, mirroring the circular tooth root. However, the highest resolution scans in our dataset revealed “trails” of alveolar bone lingual to each functional tooth in single- and multiple-rowed dicynodonts, indicating that the tooth and its accompanying socket have migrated labially through ontogeny^11^ (Figs. 5a, 6a,b, 7b,c; see also histological data).

Some tooth positions also contain the remnants of old teeth that have been partially resorbed, marking the positions of older tooth generations (Figs. 8a, 9a,b). This feature is sometimes seen in other polyphyodont amniotes^6,9,103^. These remnants are usually found within the alveolus of the functional tooth, but sometimes tooth remnants were actually captured within the pulp cavity of newer teeth (Fig. 9b). They are typically on the labial side of the functional tooth, but they are occasionally found on other sides as well. Tooth remnants are present at one out of twelve positions in the mandible of *Compsodon*, with none present in the maxilla. Similarly, *Pristerodon* has a remnant at one of ten tooth positions in the mandible (NHCC LB190) and none in the maxilla (NHCC LB231). Tooth remnants are more abundant in *Niassodon* and *Abajudon*, with remnants present at about a quarter of tooth positions in both the upper and lower jaws. It is unclear whether there are tooth remnants in the *E. tolani* specimen NHCC LB684. Because there does not seem to be a complete replacement of teeth in the multiple-rowed mandibles of *E. bathystoma* (NHCC LB816 and NHCC LB11), there are no remnants of older teeth. Instead, the older teeth are retained in the jaw, with newer teeth added lingually to create an additional row (Fig. 7b,c).

In specimens with multiple tooth rows, the labial teeth are smaller than the lingual ones, suggesting that the former erupted earlier in the animal’s life, as is the case in captorhinids^4–6^. Most of the sampled taxa also have one to three tiny teeth at the distal end of the jaw. These may be teeth that erupted early in life and were never replaced, which is also seen along the same regions of the multiple tooth rows in captorhinids^4–6^. Alternatively, these teeth may have been added to the distal end of the jaw later in the animal’s life as the jaw grew large enough to accommodate them, as is sometimes seen in modern reptiles^3^.

## Discussion

### Dynamics of tooth replacement in single-rowed dicynodonts and its implications for the evolution of multiple tooth rows in *Endothiodon*

**Figure 12:**
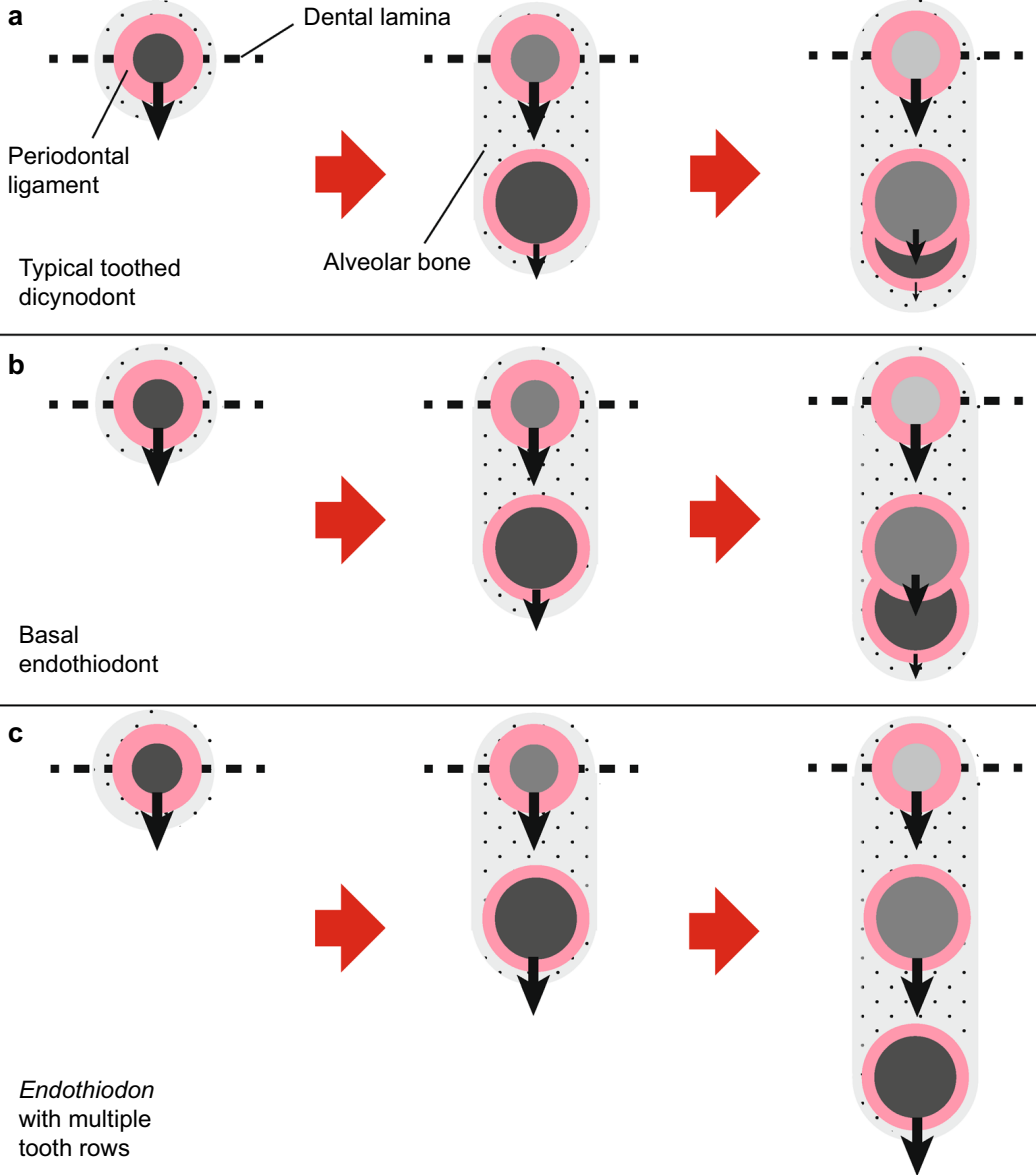
Model of multiple tooth row development in *Endothiodon bathystoma*. (**a**) Typical tooth replacement in a stemward dicynodont, in which the migration of functional teeth and their sockets (black arrows) slows over time, allowing replacement by newer teeth. (**b**) Tooth replacement in stemward endothiodonts, in which faster migration of functional teeth causes incomplete tooth replacement to happen more frequently, leaving behind tooth remnants. (**c**) Tooth emplacement in *Endothiodon* with multiple tooth rows, in which functional teeth and sockets maintain a rapid migration pace, allowing them to avoid erosion.

**Figure 13:**
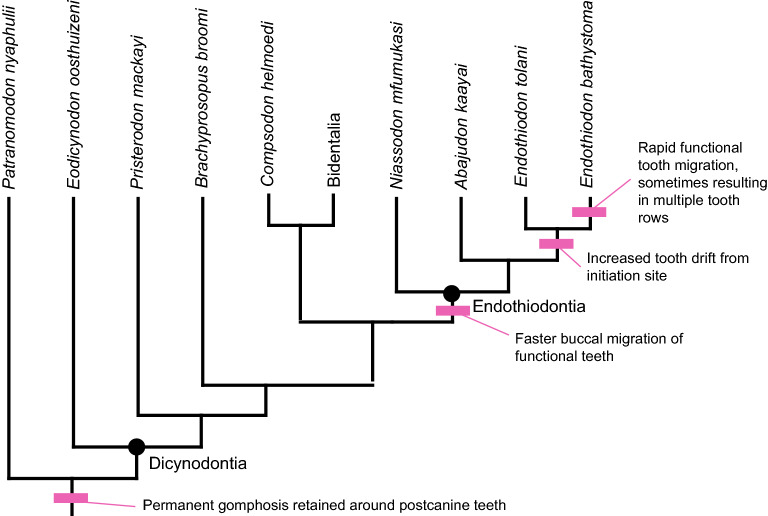
Cladogram illustrating changes in tooth replacement across stemward dicynodonts. Phylogeny based on Olroyd et al.^47^, Kammerer and Ordoñez^106^, and Angielczyk et al.^107^, but note that other topologies for *Pristerodon, Brachyprosopus*, the endothiodonts, and the emydopoids have appeared in recent phylogenetic analyses^42,108,109^.

Our CT images of single-rowed dicynodonts revealed that teeth are generally replaced lingually. The developing tooth slowly migrates labially to begin resorbing the base of the functional tooth (Fig. 12a). This process is identical to that present plesiomorphically in other amniotes^3,6^. However, the abundance of remnants of former functional teeth in the endothiodonts we examined suggests that tooth replacement was comparatively imprecise in these taxa. The migration of functional teeth must have been fast enough for the teeth to occasionally avoid complete resorption by their replacements (Fig. 12b)^9^. The labial migration of functional teeth in the endothiodonts *Niassodon* and *Abajudon* is not as extreme as that seen in some ceratopsian dinosaurs, in which multiple generations of old teeth are preserved as partially resorbed remnants within the jaws^103^, but it does seem to represent a slight increase in functional tooth migration compared to other dicynodonts (Fig. 13). This shift may have been accentuated in *Endothiodon* to allow the development of multiple tooth rows in some individuals. *Endothiodon* is also unique in its apparent deviation from the lingual replacement cycle seen in the other taxa, with its replacement cycle ranging from mesio- to distolingual (Fig. 7). This is likely the result of more rapid and complex tooth drift from the tooth initiation sites, which could cause the replacement cycle to be imprecise at multiple tooth positions.

*Abajudon* seems to be the exception to the patterns seen in the other taxa. Compared to other dicynodonts, replacement tooth initiation in *Abajudon* occurs deeper within the jaw, more generations of replacement teeth are present at some tooth positions, and more replacement teeth are present (Figs. 6b, 9b). The unique features of tooth replacement in *Abajudon* are probably related to its highly specialized dentition, in which the teeth have carinae with distinct fluting between them. The exact function of this tooth shape is unknown, but it has been hypothesized based on the rest of the feeding apparatus that these teeth were involved in holding food in place while the lower teeth sliced through it^47^. This is supported by rough similarities with the mesialmost teeth of tyrannosaurs, in which the small angle between carinae is thought to allow greater resistance to torsional forces^104^. The proposed feeding method for *Abajudon* likely would have involved heavy bending stresses that may have led to frequent tooth loss, so it would have been important to have replacement teeth immediately available. The bizarre dentition in this taxon highlights a need to thoroughly investigate the dietary range of dicynodonts, especially given recent work showing that at least some dicynodonts were not strictly herbivorous throughout all life stages^105^. In summary, the diet and/or feeding system of *Abajudon* was probably unique among dicynodonts, and this is reflected in its tooth replacement.

### Multiple tooth row development in *Endothiodon* compared to captorhinids

The multi-rowed dentition of *Endothiodon* superficially resembles the staggered, diagonal rows of teeth in the early Permian reptile *Captorhinus aguti*^6^. This clearly represents a case of evolutionary convergence, yet our CT and histological data reveal fundamental differences in the development and maintenance of multiple tooth rows in *Endothiodon* compared to captorhinids. These differences likely reflect the disparate evolutionary histories of therapsids and early reptiles. Therefore, we present a new model for multiple tooth row development outside of Reptilia, highlighting the importance of a gomphosis and tooth drift to the development of a complex dentition in *Endothiodon*.

As in the single-rowed dicynodonts and other amniotes, *Endothiodon* exhibits labial migration of newly developed teeth towards the older teeth in the early stages of the replacement cycle. The feature that differs in the multi-rowed *Endothiodon* dentitions is the spatial separation of the functional and replacement teeth. As in the multiple tooth rows of captorhinids, the newest, largest teeth form along the lingual margin of the jaws, which indicates that the odontogenic organ, the dental lamina, resides along this lingual margin (Fig. 12). In both captorhinids and *Endothiodon*, the initiation of new teeth occurred slowly. The dramatic size difference between teeth in different rows in NHCC LB11 indicates that the addition of tooth rows took place over an extended period of time, which is consistent with the fairly slow replacement rates observed in other therapsids^69^. This is comparable to the size discrepancies between teeth in different rows in captorhinids^6^. Hadrosaurs and other dental battery-bearing dinosaurs show the opposite: rapid tooth replacement rates with similar-sized tooth generations, and continuous vertical eruption of teeth from deep within the jaw^8,9^.

The development of multiple tooth rows like those of *Endothiodon* and captorhinids requires relatively slow tooth initiation rates. These provided older teeth with adequate time to migrate away from the sites of tooth initiation and avoid replacement. The migratory mechanisms in captorhinids and *Endothiodon*, however, are different. Like nearly all other Paleozoic reptiles, captorhinid teeth were ankylosed (fused) to the jaws upon completing eruption and were thus incapable of any post-eruptive drift^5,6^. Instead, the dentaries and maxillae grew asymmetrically; old bone was resorbed along the labial surface, and new bone was deposited along the lingual margin. The net effect was a labiolingual conveyor belt that carried teeth across the jaws. As the jaws continued to grow in mesiodistal length, the teeth became staggered into diagonal rows^6^.

In *Endothiodon*, the teeth were not fused in place, but were held within their alveoli by a non-mineralized periodontal ligament (a gomphosis) (Figs. 2, 3, 7a,b, 8, 9, 10). A gomphosis-type tooth attachment is very common among therapsids, including mammals^69,100^. This pliable tooth-bone interface allows erupted teeth to gradually migrate along the jaws. In modern mammals, teeth and their sockets can migrate via resorption along the leading edge of the tooth and socket, and deposition of alveolar bone along the trailing edge, maintaining the ligamentous attachment of the tooth to the jaw in the process^100^. This process leaves a trail of alveolar bone behind each migrating tooth and marks the direction of this drift. Evidence for this mode of tooth drift is also seen in other therapsids^69^. The trails of alveolar bone lingual to each functional tooth in our specimens indicate that the teeth and sockets drifted labially in many toothed dicynodonts, independently of the jawbone (Figs. 2c, 5a(iii), 6a(iii), 7b(iii),c(iii), 8a(iii), 9a(iii),b(iii)). In most of these dicynodonts, the drift of each functional tooth was subtle, allowing a newer tooth to fully erode and replace it. However, in *Endothiodon* specimens with multiple tooth rows, the functional teeth continued to migrate fast enough to avoid resorption normally caused by the encroaching replacement teeth, allowing some individuals to retain multiple tooth generations. This is especially clear in the partially-multi-rowed specimen NHCC LB816, in which labial teeth have been slightly eroded by more lingual teeth, indicating that the number of tooth rows depends on the rate of tooth drift (Fig. 7b). The cause of this coordinated labial tooth drift is still unclear. However, as in captorhinids, the continued mesiodistal growth of the jaws likely staggered the teeth into the diagonal rows through ontogeny.

### Differences between the maxilla and mandible

In *Endothiodon*, it is much more common to find multiple tooth rows in mandibles than in maxillae. When maxillae are found with multiple rows, the ‘rows’ are generally only a few tooth positions with an extra tooth, unlike the more conspicuous multiple rows often found in the mandibles^44,46,55^. Some authors have even questioned the presence of multiple tooth rows in the maxillae of *Endothiodon,* positing that the extra teeth were broken off from the lower jaw^44^. Our CT scans demonstrate that at least some specimens of *E. bathystoma* genuinely contain multiple rows of teeth in the maxilla (Fig. 10b). We investigated possible developmental explanations for the difference in tooth row morphology by comparing mandibles and maxillae of *Endothiodon.* The multi-rowed *Endothiodon* maxilla NMT RB23 exhibits some erosion of the labial teeth by the more lingual teeth in positions where there are multiple teeth erupted, similar to what is seen in the partially-multi-rowed dentary NHCC LB816. This suggests that there was less migration of the functional teeth in the multiple-rowed upper jaws than in the lower jaws, which is likely the developmental reason that multiple tooth rows are less common in the upper jaws of *Endothiodon.*

The limited tooth drift in the maxilla of *Endothiodon* could reflect a lack of selective pressure to develop multiple tooth rows in the upper jaw. In dicynodonts, the palatines are expanded to form pads that were likely covered in keratin^46^. The palatine pads may have prevented the maxillae from expanding medially and holding more tooth rows. Additionally, the unique feeding system of *Endothiodon* was unlikely to benefit from having multiple rows of teeth on the maxilla. Unlike in captorhinids, tooth-to-tooth occlusion was not the sole component of chewing in *Endothiodon*. The chewing cycle of *Endothiodon* has been reconstructed to involve posterolateral movement of the mandible to slide the long mandibular tooth row(s) against the palatine pads^46^. Extra tooth rows would have provided more continuous contact between the mandibular teeth and the palatine pads in addition to slicing against the maxillary teeth. There was no such surface for the maxillary teeth to interact with, so there would have been little benefit to having multiple tooth rows in the maxilla. This hypothesized function is supported by the fact that the mandibles of *Endothiodon* typically have more rows at the distal end than at the mesial end, as the distalmost teeth are the ones that would contact the palatine pads. *Endothiodon* maxillae with multiple tooth rows show no major difference mesiodistally. Rare *Endothiodon* specimens with extra teeth on the upper jaw may be the result of random variation in tooth drift in the maxilla.

There are two anatomical differences between the maxillary and mandibular tooth rows of single-rowed dicynodonts as well. One is that the periodontal space is consistently wider in the mandible than in the maxilla. The other is that there are generally fewer teeth in the upper jaw than in the lower jaw. Tooth replacement itself shows little difference between the mandible and maxilla of most of the sampled taxa, but there are some noteworthy differences in *Abajudon* and *Endothiodon* (Figs. 6b, 7). In *Abajudon*, the geometry of tooth replacement differs between the two jaws: the mandible has a shallower site of initiation for tooth development, and erosion of the functional tooth begins deeper on the functional tooth root. *Endothiodon tolani* shows mesiodistal differences in the number of replacement teeth present in only the mandible. These two taxa seem to have some level of decoupling of tooth replacement in their upper and lower jaws. It is uncertain whether or not this decoupling occurred ancestrally for these two taxa, but a plesiomorphic difference in tooth replacement between the mandible and maxilla may have contributed to the evolution of multiple tooth rows on the mandible of *E. bathystoma.*

### The evolution of a gomphosis in dicynodonts

Our comparative analysis of tooth attachment in *Endothiodon* and its relatives provides crucial context for how *E. bathystoma* evolved multiple rows of teeth. Based on our taxonomic sample, it would appear that *Endothiodon* inherited its ligamentous tooth attachment from a common ancestor, rather than evolving this mode of attachment to assist in the development and maintenance of multiple tooth rows. In addition, the presence of a permanent gomphosis in the ‘postcanine’ teeth of all of the dicynodonts we examined, as well as the non-dicynodont anomodont outgroup taxon *Patranomodon*, has further implications for the evolution of tooth attachment tissues within Synapsida. LeBlanc et al.^69^ demonstrated that the stereotypically mammalian gomphosis evolved much earlier within Therapsida and may have gone through repeated reversals between fused and ligament-based tooth attachment modes within each of the major therapsid lineages. Their histological sample of dicynodonts was limited to two species with reduced dentitions. They found that the tusks of *Diictodon* were fused to the maxilla, as is common for many early therapsids and “pelycosaurs”^69^. However, the same study found that the tusks of *Lystrosaurus*, a later-diverging dicynodont, retained a permanent gomphosis. The limited taxonomic sampling, which did not include any dicynodonts with ‘postcanines’, meant that they could only speculate on the evolution of tooth attachment within dicynodonts. Though the relationships between stemward dicynodonts are still largely unresolved^47,99^, our sample likely represents three separate lineages near the base of the dicynodont phylogeny and a non-dicynodont anomodont with a more normal marginal dentition, shedding new light on dicynodont dental evolution. The retention of a gomphosis in fully mature ‘postcanine’ teeth of all of our study taxa indicates that a permanent gomphosis around the ‘postcanines’ is likely the ancestral condition for all of Dicynodontia (Fig. 13). The tooth attachment geometry in anomodonts stemward of *Patranomodon* is currently unknown, although scans of the venyukovioid *Suminia* suggest that it does not have a gomphosis (JB personal observation), and the distribution of a permanent gomphosis around dicynodont tusks is currently under study (Megan R. Whitney, personal communication, 2020). These findings complement those of LeBlanc et al.^69^ and Whitney et al.^72,98^, further supporting the acquisition of a permanent gomphosis early in therapsid evolution through heterochronic changes in the degrees of mineralization of alveolar bone and the periodontal ligament.

## Conclusions

1. A permanent gomphosis is present in ‘postcanine’ teeth of stemward dicynodonts and the non-dicynodont anomodont *Patranomodon*, making this feature the likely plesiomorphic condition for Dicynodontia.
2. Stemward dicynodonts show the typical alternating tooth replacement seen in most amniotes. Functional tooth migration seems to increase within Endothiodontia, which probably contributed to the acquisition of multiple tooth rows in *Endothiodon*.
3. The multiple tooth rows of some specimens of *Endothiodon bathystoma* developed via rapid labial migration of the functional teeth and their sockets, allowing the teeth to avoid erosion by replacement teeth. This is the first time such a method of multiple tooth row development has been documented in tetrapods.
4. Multiple tooth rows in *Endothiodon* are more commonly seen in the lower jaw than the upper jaw because the maxillary functional teeth exhibit slightly less labial migration than teeth in the mandible. Other stemward dicynodonts show evidence of decoupling of tooth replacement in the upper and lower jaws, which may have contributed to the differences in migration seen in *Endothiodon*.
5. The additional tooth rows at the distal end of the dentary in *Endothiodon* likely served to provide continuous contact between the mandibular teeth and the palatine pads during a posterolateral chewing stroke. The rarity of maxillae with multiple tooth rows in this taxon likely reflects the absence of a similarly wide surface on the dentary for the occlusion of maxillary teeth.
